# Public good‐driven release of heterogeneous resources leads to genotypic diversification of an isogenic yeast population

**DOI:** 10.1111/evo.14646

**Published:** 2022-10-13

**Authors:** Anjali Mahilkar, Prachitha Nagendra, Phaniendra Alugoju, Rajeshkannan E, Supreet Saini

**Affiliations:** ^1^ Department of Chemical Engineering Indian Institute of Technology Bombay Mumbai 400076 India

**Keywords:** Genetic divergence, melibiose, *Saccharomyces cerevisiae*, sympatry

## Abstract

Understanding the basis of biological diversity remains a central problem in evolutionary biology. Using microbial systems, adaptive diversification has been studied in (a) spatially heterogeneous environments, (b) temporally segregated resources, and (c) resource specialization in a homogeneous environment. However, it is not well understood how adaptive diversification can take place in a homogeneous environment containing a single resource. Starting from an isogenic population of yeast *Saccharomyces cerevisiae*, we report rapid adaptive diversification, when propagated in an environment containing melibiose as the carbon source. The diversification is driven due to a public good enzyme α‐galactosidase, which hydrolyzes melibiose into glucose and galactose. The diversification is driven by mutations at a single locus, in the GAL3 gene in the *S. cerevisiae* GAL/MEL regulon. We show that metabolic co‐operation involving public resources could be an important mode of generating biological diversity. Our study demonstrates sympatric diversification of yeast starting from an isogenic population and provides detailed mechanistic insights into the factors and conditions responsible for generating and maintaining the population diversity.

Adaptive divergence of existing populations may lead to formation of new species and forms the basis of ecological diversity of life forms. Populations can diverge when specializing toward distinct niches and/or resources. Metabolic specialization is a common mode that can lead to diversification of an isogenic population. Adaptive divergence has been observed when diversification leads to distinct genotypes to (a) occupy the different available niches (Rainey and Travisano 1998; Borer et al. 2018; Blake et al. 2021), (b) occupy new niches created by the population (Helling et al. 1987; Laland et al. 1999; Friesen et al. 2004; Saxer et al. 2010), or (c) occupy novel, previously unavailable niches via evolution of metabolic innovation (Cooper and Lenski 2000; Blount et al. 2008). In these cases, such specialization has been observed because of acquisition of a relatively small number of mutations (McDonald et al. 2009; Ferguson et al. 2013; Plucain et al. 2014). The repeated observation of emergence of specialists and the relatively easy route in the sequence space facilitating this transition indicates that metabolic specialization is likely an important ecologically relevant mode explaining genetic diversity.

Most of these studies, generally done in spatially structured populations, suggest diversification is driven by resources competition in distinct niches providing ecological opportunities. Such diversification was absent in a well‐mixed homogenous environment (Rainey and Travisano 1998). These studies suggest that competitive trade‐offs and ecological opportunity are essential to cause diversification (reviewed in Rainey et al. 2000). However, little is known about the fundamental rules and conditions under which such diversification of isogenic population takes place in a well‐mixed environment (Spencer et al. 2007). A link between phenotypic divergence leading to distinct genotypes is absent in such an environment.

Stable diversification of population depends on the dynamics of fitness relationship among the subpopulations. Coexistence of multiple genotypes in an environment can also be facilitated by metabolic trade (Pande et al. 2014; Ziesack et al. 2019). It has been demonstrated experimentally and theoretically that an auxotrophic pair of genotype/species, trading essential metabolites, can grow faster than the prototroph parent (Pande et al. 2014; Dutta and Saini 2020). Although theory explains this observation, how, starting from an isogenic prototrophic population, we can achieve a population split to take place is not well understood (Dutta and Saini 2021).

One particular manifestation of metabolic trade between two or more species is emergence of a cheater population (Smith and Schuster 2019). In such a context, some members of an isogenic population pay a cost for production of a public resource. This leads to emergence of cheaters in the population, which do not contribute toward production of the public resource, but gain from benefits from the resource produced by co‐operators in the population. Because the cheaters have a higher fitness than the co‐operators, but cannot survive in the absence of co‐operators, a stable coexistence arises (Perez‐Escudero and Gore 2016; Pollak et al. 2016). Presence of cheaters in an environment has also been shown to have a positive effect in preserving biodiversity in an unstructured space competition experiment between bacteria (Leinweber et al. 2017). This is because presence of cheaters decreases the fitness of the co‐operators, thus allowing other species to not be eliminated in the resulting environment. However, effects of cheaters on being able to influence the fitness of co‐operators are likely to be dependent on the precise environment in which fitness is tested (Sexton and Schuster 2017).

The impact of co‐operation and cheating in dictating structures of population has also been investigated using game theory. These studies have demonstrated that if interactions between participating species/genotypes can be represented via Hawk‐Dove or/and Snowdrift games, a stable coexistence arises (Gore et al. 2009; Chen et al. 2017). This game‐like representation was applied to *Pseudomonas* and *Klebsiella*, and coexistence was shown on spatial and uniform environments (Zhao et al. 2019).

In yeast, when feeding on a nonsimple disaccharide sugar sucrose, the population structure influences the fate of the population (collapse or coexistence) (Allen and Nowak 2013; Sanchez and Gore 2013). Hydrolysis of sucrose leads to release of its monosaccharide components—glucose and fructose. Interestingly, the hydrolysis takes places in the periplasm, leading to a small fraction (∼0.01) of the hydrolyzed sugars to diffuse back into the cell and the rest available as a public resource. The dynamics of growth of the two participating genotypes (co‐operator and cheater) in this case is dictated by the initial frequency of the participating genotypes in the population (Prajapat et al. 2016). Growth on sucrose as a carbon source has two characteristic features. First, growth dynamics of yeast on glucose and fructose are quite similar, particularly in nonfermenting conditions (Berthels et al. 2004; Marques et al. 2016). Second, a large (but not 100%) fraction of the total resource (the two hydrolyzed monosaccharides) is made available as a public resource.

In this work, we use melibiose as the complex metabolic environment to determine adaptive diversification because of its unique mechanism of using melibiose. Several *S. cerevisiae* strains can grow on melibiose as a carbon source. Melibiose is a disaccharide of glucose and galactose, and is hydrolyzed into its constituent monosaccharides by the action of an extracellular α‐galactosidase enzyme encoded by the gene *MEL1* (Johnston and Hopper 1982). Of the two monosaccharides, *S. cerevisiae* consumes glucose rapidly and preferentially over galactose. Galactose use, controlled by the GAL regulon, is extremely sensitive to the amounts of glucose in the environment (Piskur et al. 2006) (Fig. 1). Expression of genes necessary for galactose use is activated by the Gal4p transcription factor (Keegan et al. 1986; Ma and Ptashne 1987). In the absence of galactose, Gal4p is sequestered by Gal80p. Thus, promoters of the GAL regulon are in the OFF state. The Gal80p‐dependent repression is relieved in the presence of galactose, when the signal transducer Gal3p or Gal1p binds Gal80p, hence freeing up Gal4p to activate gene expression from promoters of the GAL regulon. Gal1p, in addition to its role as a signal transducer, also acts as a kinase, necessary for galactose use in the cell (Bhat and Murthy 2001; Rubio‐Texeira 2005). Interestingly, MEL1 is also a part of the GAL/MEL regulon, and once expressed, Mel1p is secreted into the extracellular media.

**Figure 1:**
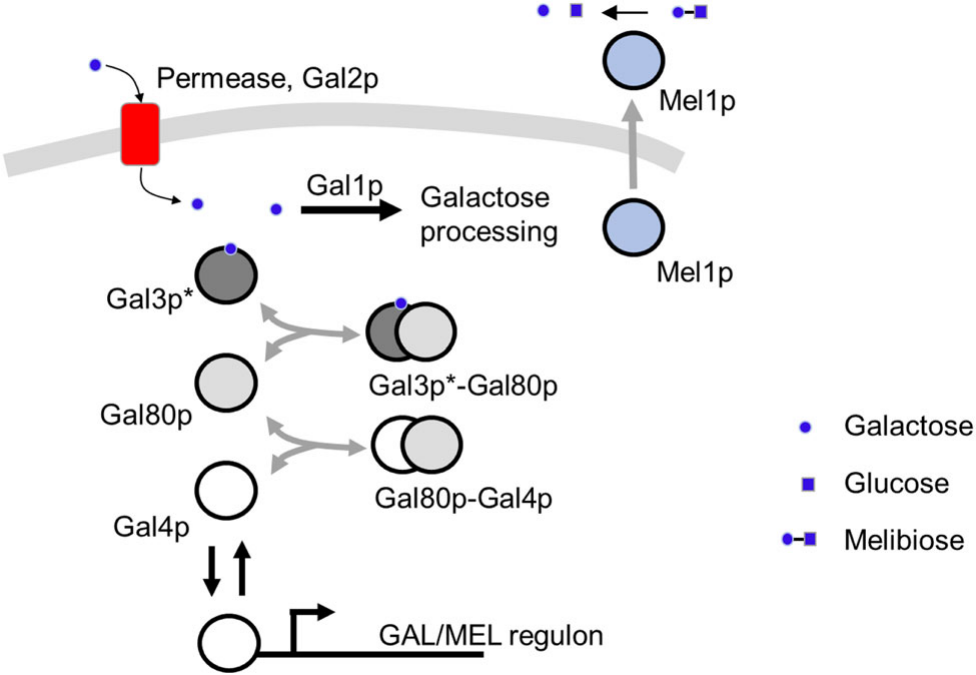
GAL/MEL network. Genes in the GAL/MEL regulon are activated by the transcription factor Gal4p. Gal4p is sequestered by the protein Gal80p, which binds to Gal4p to form the Gal80p‐Gal4p complex. Gal80p‐dependent repression is relieved when Gal3p/Gal1p binds Gal80p, thus freeing Gal4p for activation of the network. Galactose is imported into the cell via the permease Gal2p. Intracellular galactose is processed in a metabolic chain. Also part of the GAL/MEL regulon is Mel1p. Mel1p is released into the extracellular media, where it hydrolyzes melibiose into glucose and galactose. Regulation of the GAL/MEL regulon is reviewed in Bhat and Iyer (2009).

The melibiose use system is different from the sucrose use system in three aspects. First, the disaccharide is hydrolyzed in the extracellular environment. Hence, all of glucose and galactose so produced are a public resource. Second, kinetics of growth (duration of lag phase and growth rate) of *S. cerevisiae* on glucose and galactose are quite different from each other (Fig. 2a). Third, following hydrolysis of melibiose the resulting monosaccharides each have antagonistic regulatory effect on expression of MEL1. Although galactose induces the expression of Mel1p, glucose represses expression of Mel1p due to catabolite repression.

**Figure 2:**
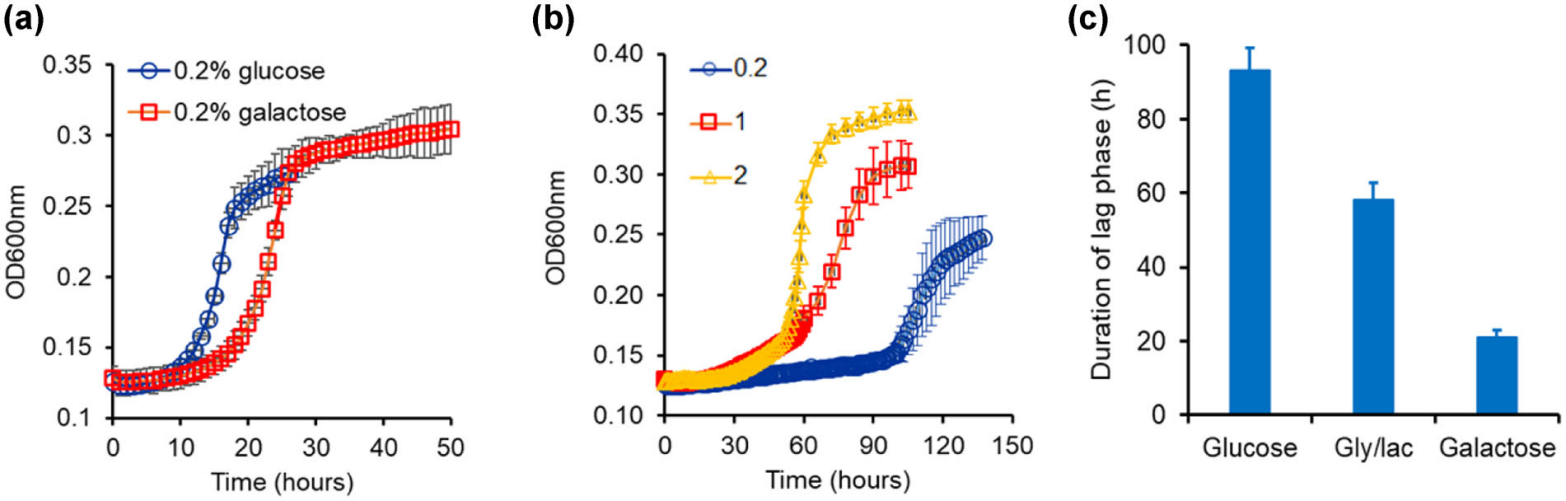
(a) Growth kinetics of *Saccharomyces cerevisiae* in glucose and galactose. Growth rate on glucose is 0.0438 h^−1^ and that in galactose is 0.0406 h^−1^. The duration of lag in glucose is 11 h and that in galactose is 17 h. Growth rate and the duration of lag were determined by taking log of the OD600 values and plotting against time. The slope of the linear part of the trajectory is calculated as the growth rate of the culture. The intercept of the linear part in this curve with the log of OD600 at *t* = 0 was used as a measure of the duration of the lag phase. (b) Growth kinetics of *S. cerevisiae* in different melibiose concentrations. Cells were grown in noninducing nonrepressing conditions (gly/lac) and then subcultured in melibiose‐containing media. Average of three runs and the standard deviation is reported. The duration of the lag phase was 42, 58, and 98 h for cultures grown at 2%, 1%, and 0.2 % melibiose, respectively. The corresponding values of the growth rate were found to be 0.0385, 0.0145, and 0.01 h^−1^. (c) Duration of the lag phase depends on the pre‐inoculum conditions. Lag phase duration in melibiose is longest when the cells are brought from glucose and shortest when the cells are introduced into melibiose media from galactose containing media. All experiments were performed in triplicate, and average and standard deviation are reported.

In this work, we use the melibiose use system in *S. cerevisiae* as a model system in which processing of a resource (melibiose) by a public good (Mel1p) leads to the population splitting into two distinct genotypes. We demonstrate that during growth on melibiose, metabolic heterogeneity is observed in an isogenic population during the exponential phase of growth. By serial subculturing to maintain the cells in the state of heterogeneity, growth for a few hundred generations leads to the population genetically splitting into two distinct phenotypes. The two phenotypes/genotypes are distinguished by the colony size on solid melibiose media, and also by growth dynamics in glucose and galactose. Sequencing results show that the coexistence is maintained via polymorphism at the GAL3 locus. Overall, these results show that simple genetic changes can facilitate diversification of an isogenic population into two distinct genotypes even in spatially unstructured environments. This diversification is driven by dynamic release of a public good (Mel1p), which leads to release of heterogeneous carbon sources (glucose and galactose).

## Materials and Methods

### STRAIN USED

A SC644 diploid (a/α) strain was used in this study.

### GROWTH KINETICS

Glycerol‐lactate pregrown strains were plated onto Synthetic Complete Media (SCM) agar plates (containing sugar concentration as defined). The plates were incubated at 30°C for 3–4 days, after which, random colonies were selected and passaged twice in appropriate media (as described in *Results*). The resulting cultures were then washed with SCM and then growth curves were initiated with an initial optical density of 0.1 in SCM containing sugar(s) at an appropriate concentration as described. Three independent replicates of each condition were transferred to a 96‐well plate and Optical density (OD) was measured periodically until the cultures reach stationary phase. The plates were overlaid with a *Breathe Easy* membranes (Sigma) to prevent evaporation. To calculate the growth rate, log(OD), in the exponential phase of growth, was plotted against time. The slope of the straight‐line fit was calculated as the growth rate of the strain. The *x*‐axis value (time) where this straight line intercepts *y* = log(initial OD) was taken to be the duration of the lag phase of growth.

### EXPERIMENTS WITH 2‐DEOXY GALACTOSE

Ancestral and evolved strains were incubated in Gly/lac medium and incubate for 48–72 h. The gly/lac pregrown cultures were inoculated into 5 mL of CSM containing melibiose and incubated at 30°C. Cells were harvested in exponential phase, diluted with PBS, and thereafter, plated onto gly/lac plates containing different concentrations (0%, 0.3 μM, and 0.6 μM) of 2‐deoxygalactose (2DG). Plates were incubated at 30°C for 3–4 days. The number of colonies that grew on gly/lac plates (0% 2DG) and those on plates containing 2DG was counted and the percentage of Gal‐positive cells was calculated for both ancestral and evolved strains.

### EVOLUTION EXPERIMENT

Diploid (**a/α**) SC644 (*MATa MEL1ade1 ile trp1‐HIII ura3‐52*) strain was used for the evolution experiments to demonstrate speciation events. A single colony was grown in liquid CSM galactose medium (inducing medium) for 24 h at 30°C on a rotary shaker at 250 rpm. To start the evolution experiment, 50 μL of this overnight culture was diluted 1:100 into fresh media (CSM containing melibiose). Three parallel populations lines were evolved in CSM containing 2% melibiose. Populations were propagated in 5 mL of liquid medium (30°C, 250 rpm) within 25 × 150 mm borosilicate tubes. After every 24 h of growth (∼6.6 generations/day), 50 μL of each culture was transferred to 5 mL of fresh medium containing respective concentrations of melibiose daily for up to 400 generations.

### COLONY SIZE ANALYSIS

Plate images were taken in *UVITECH* gel documentation unit in white light at an exposure of 800 ms, 3× zoom, and 900 focus using the *Essential V6* software. The colony size was measured using CellProfiler 3.1.8 using the automated pipeline described in Bray et al. (2015). All colonies between pixels 6 and 95 were considered during the analysis.

### SEQUENCING

Large and small colonies of evolved line 1 were sporulated, and the four haploids were isolated. For each of the four haploids, sequencing of the following promoter regions and the coding sequences was done. For GAL1, primers 5ʹ – TTA ACT GCT CAT TGC TAT AT – 3ʹ and 5ʹ – AAA AGA AGT ATA CTT ATA AT ‐ 3ʹ were used; for GAL3, primers 5ʹ – GCT TTT ACT ATT ATC TTC TA – 3ʹ and 5ʹ – TTG TTC GTA CAA ACA AGT AC – 3ʹ were used; for GAL4, primers 5ʹ – GGA CCC TGA CGG CGA CAC AG – 3ʹ and 5ʹ – CAT TTT ACT CTT TTT TTG GG – 3ʹ were used; for GAL80, primers 5ʹ – CAG ATG GAA TCC CTT CCA TA – 3ʹ and 5ʹ – GCA CTG GGG GCC AAG CAC AG – 3ʹ were used; and for MEL1, primers 5ʹ – GTC GAC TTC TAA GTA AAC AC – 3ʹ and 5ʹ – TGC TTT GCT CAA CAA TAA GC – 3ʹ were used. MEL1 sequence was taken from a previous report (Liljestrom 1985). For sequencing, individual colonies of respective strains were collected from Yeast extract Peptone Dextrose (YPD) plates, and allowed to grow for 6–8 hours in liquid YPD media at 30°C. The cells were harvested, and their genomic DNA was isolated. The five DNA sequences were amplified by PCR. Sequencing was done by Eurofins Scientific.

### GAL3 CLONING

The mutant GAL3 allele was amplified using the primers pSC034 (CGA GTC GAA TTC AAT ACA AAC GTT CCA ATA) and pSC038 (AAG CTT GAG TAA ACT TTT AAT ATT TAA) from the large colony of evolved E1 line from the melibiose plate and cloned into the plasmid pYJM (Murthy and Jayadeva Bhat 2000) between the *Eco*RI and *Hin*dIII cut‐sites. The ancestral allele was amplified from the ancestor using the same primer set and cloned as described above. The resulting plasmids pYJM‐GAL3* and pYJM‐GAL3 and pYJM were transformed into BY4742 ΔGAL3::KanMX4 (Euroscraf) strain.

### COMPETITION EXPERIMENT

BY4743 and the ancestral strain were grown separately in noninducing nonrepressing conditions till saturation. Roughly 10^6^ cells of each genotype were transferred to a tube containing 1% melibiose, and allowed to grow for 24 h. The culture (at *t* = 0 at the beginning of the experiment, and at *t* = 24 h) was plated on YPD and ura‐ trp‐ double dropout plates to quantify the relative frequency of the two genotypes. The relative fitness was calculated using the formula below, as described in Gagneux et al. (2006):

$$\mathrm{Relative}\;\mathrm{fitness},\;f\;=\;\frac{\ln\left[\frac{\mathrm{anc}\;\mathrm{at}\;t=24\;h}{\mathrm{anc}\;\mathrm{at}\;t=0}\right]}{\ln\left[\frac{\mathrm{Evol}\;\mathrm{at}\;t=24\;h}{\mathrm{Evol}\;\mathrm{at}\;t=0}\right]},$$

where anc. at *t* = 24 h refers to the CFU count of the ancestral strain at time 24 h. The relative fitness of the evolved strain with respect to the strain BY4743 was calculated similarly.

### α‐GALACTOSIDASE ENZYME ACTIVITY

Extracellular α‐galactosidase assay was performed to determine the expression level of the Mel1p, as described previously (Diep et al. 2006). Yeast strains were grown in synthetic complete medium containing gly/lac up to saturation. The cultures were then subcultured in synthetic complete media containing 2% galactose to an initial OD of 0.05. The cultures were then allowed to grow till an OD of 1.00. A volume of 1 mL of each culture was centrifuged and extracellular α‐galactosidase activity of the supernatant was determined as follows. A total of 120 μL of the supernatant was mixed with 360 μL of assay buffer (2 volumes of 0.5 m sodium acetate, pH 4.5, and 1 volume of 100 mm p‐nitrophenyl α‐d‐galactopyranoside [Sigma]). The reaction was incubated at 30°C for 5 h and terminated by adding 520 μL of stop buffer (1 M sodium carbonate). Enzyme amounts were then determined by measuring the absorbance at 410 nm. Triplicate samples were taken for the analysis and results represent average of at least three independent experiments with standard deviation.

### CO‐CULTURE EXPERIMENTS

SUC2 was deleted from the strain ΔGAL3::KanMX4 using the primers 5ʹ – CAA GCA AAA CAA AAA GCT TTT CTT TTC ACT AAC GTA TAT GAT GCT TTT GCG CAG GTC GAC AAC CCT TAA T – 3ʹ and 5ʹ –TTT AGA ATG GCT TTT GAA AAA AAT AAA AAA GAC AAT AAG TTT TAT AAC CTA GTG GAT CTG ATA TCA CCT A – 3ʹ (Check primers CTCTTGTTCTTGTGCTTTTT and ATTCTTTGAAATCATAAAGT). The resulting strain, ΔGAL3 ΔSUC2, was transformed with pYJM‐GAL3. The strain ΔGAL3 was transformed with pYJM‐GAL3*. The two strains were inoculated from a single colony into gly/lac media and allowed to grow for 48 h. Cells from the two cultures were mixed into a melibiose‐containing tube, and allowed to grow for 48 h. Cells from the culture were then spread on a YPD plate for single colonies. The single colonies were then streaked on Hygromycin (200 μg/mL) containing YPD plates to quantify the frequency of each genotype. A minimum of 500 colonies were streaked for each culture experiment. All experiments were done three times independently.

### COST‐BENEFIT MODELING

In the generalist strategy, the cells can acquire mutations that eliminate glucose‐dependent repression of galactose genes. In such a scenario, each individual cell uses glucose and galactose simultaneously, while exporting Mel1p. Because all cells behave metabolically identically in this strategy, we call this a generalist strategy. Each cell consumes equal amounts of glucose and galactose, which is equal to the amount of melibiose broken down by it. We start with modeling the benefit gained by a cell, in terms of fitness, as a function of sugar concentration.

At steady state in a chemostat, let the rate of melibiose hydrolysis per time by Mel1p released per cell be *k*. Therefore, the amount of glucose and galactose produced by hydrolysis to be consumed per time is also *k* each. Let us assume that the benefit conferred to the cell upon metabolizing one molecule of glucose (or galactose) is *b*.

We assume that the benefit conferred to a cell upon consumption of *k* molecules of a substrate is of the following form:

(1) $$b=b_{\max}\frac{k}{K_{m}+k},$$

where *b*_max_ is the maximum possible benefit conferred as *k* approaches values much greater than half‐maximum substrate amounts *K*_m_. In both the strategies, we place the constraint that the maximum cellular flux that can be processed in a cell is *k*_max_.

Because in the generalist case, the total number of molecules of the substrate consumed per time is *2k*, the benefit conferred to the cell can be written as

(2) $$\mathrm{Benefit},\;b=b_{\max}\frac{2k}{K_{m}+2k}.$$

On the other hand, use of carbon substrate requires investment in the form of synthesis of appropriate enzymes and commitment of cellular resources (like, ribosomes, amino acids) toward enzyme synthesis. This cost is proportional to the number of substrate molecules being used. Moreover, suppose each enzyme molecule in the pathway processes *a* substrate molecules per time, then the total cost can be represented as

(3) $$\mathrm{Cost},\;c=\frac{2k}{a_{\mathrm{glu}}}\;c_{\mathrm{glu}}^{o}+\;\frac{k}{a_{\mathrm{gal}}}\;c_{\mathrm{gal}}^{o},$$

where *a*_glu_ is the number of molecules processed per enzyme molecule in the glucose use pathway, and *a*_gal_ is the number of substrate molecules per enzyme molecule in the galactose to glucose‐6‐phophate pathway. Note that from galactose‐6‐pathway, the processing of carbon is via a common metabolic path, and hence, *2k* molecules are added in the cost term.

The fitness of the cell is therefore given as

(4) $$\mathrm{Fitness},\;f=b_{\max}\frac{2k}{K_{m}+2k}-\;\frac{2k}{a_{\mathrm{glu}}}\;c_{\mathrm{glu}}^{o}-\;\frac{k}{a_{\mathrm{gal}}}\;c_{\mathrm{gal}}^{o}.$$

Alternatively, the population may evolve such that different cells adopt different strategies, with the goal that the fitness of the population is maximized. Such a strategy, where two or more different metabolic states optimize fitness, is referred to as a specialist strategy.

In this strategy, one fraction of the population hydrolyzes melibiose into glucose and galactose. A fraction of the population is a cheater population, which does not contribute toward Mel1p production, but instead uses glucose released from melibiose hydrolysis. As a result, the Mel1p‐producing fraction of the population consumes galactose for growth, and keep producing Mel1p for continued hydrolysis of melibiose. The cheaters are preserved in the population because of their higher fitness, and the galactose users (ancestral cells) are maintained because they are essential for melibiose hydrolysis. Such a coexistence of two distinct genotypes has been observed in microbial system.

In a population of size *N*, let the fraction of co‐operators (ancestral cells) be *x*. Therefore, the fraction of cheaters is (1 – *x*). Let the rate of hydrolysis be *k*, due to Mel1p released by each co‐operator cell. Therefore, total breakdown of melibiose per time = (*Nx*)*k*, and consequently, the amount of glucose and galactose released per time also equals (*Nx*)*k*.

Assuming that the galactose users consume galactose, the amount of galactose per cell is *k*. Similarly, assuming that the cheaters consume glucose, the amount of glucose per cell is $\frac{kx}{1-x}$.

Therefore, as defined earlier, the fitness of co‐operators is

(5) $$f_{\mathrm{coop}}=b_{\max}\frac{k}{K_{m}+k}-\frac{k}{a_{\mathrm{gal}}}c_{\mathrm{gal}}^{o}-\frac{k}{a_{\mathrm{glu}}}c_{\mathrm{glu}}^{o}.$$

Similarly, the fitness of the cheater cells can be quantified as

(6) $$f_{\mathrm{cheapters}}=b_{\max}\frac{\frac{kx}{1-x}}{K_{m}+\;\frac{kx}{1-x}}\;-\;\frac{\frac{kx}{1-x}}{a_{\mathrm{glu}}}\;c_{\mathrm{glu}}^{o}.$$

Now, for coexistence of co‐operators and cheaters, the two should have equal fitness. All the parameters described above are inherent cellular properties, except for the hydrolysis rate *k*. It is a function of (1) the production rate of α‐galactosidase, *p*_o_, (2) the degradation rate of α‐galactosidase, *k*_d_, and (c) processing rate of by α‐galactosidase per enzyme, which is dependent on the concentration of melibiose, *α*.

The enzyme dynamics in the extracellular environment can be described as

(7) $$\;\frac{dE}{dt}=p_{o}-k_{d}E,$$

where *E* is the enzyme concentration in the environment. At steady state, $E=\frac{p_{o}}{k_{d}}$. Thefore, the hydrolysis rate, *k* = *Eα*.

## Results

### DYNAMICS OF GROWTH IN MELIBIOSE

Melibiose is a disaccharide of glucose and galactose. Expression of the enzyme Mel1p, which splits melibiose into the constituent monosaccharides, is induced by the transcription factor Gal4p, in the presence of galactose. Presence of glucose in the media represses expression of the GAL regulon, as well as that of Mel1p (Post‐Beittenmiller et al. 1984). Logically, this situation is identical to that of lactose use in *E. coli*, with two key differences. First, in *E. coli*, the disaccharide is split into the monosaccharides inside the cell (Ozbudak et al. 2004), whereas in the case of *S. cerevisiae*, melibiose is hydrolyzed outside the cell. Second, in *E. coli*, the inducer molecule is the disaccharide, whereas the inducer for gene expression is the monosaccharide (galactose) in *S. cerevisiae*.

Growth on melibiose takes place after a long lag phase. The dynamics of growth on increasing concentrations of melibiose is as shown in Figure 2b. The lag duration and the rate of growth are tunable parameters, and decrease and increase, respectively, with increasing melibiose concentration. As compared with growth on equal amounts of carbon, the lag durations associated with glucose and galactose are much smaller compared to that of melibiose.

The duration of the lag is also a function of the initial state of the system, that is, the metabolic state from which they are introduced into melibiose. When cells from (a) an OFF state (in glucose), (b) an ON state (in galactose), and (c) neutral state (glycerol‐lactate) with respect to GAL gene induction are introduced in media containing melibiose, the duration of the lag is strongly dependent on the environmental history of the cells. Cells transitioned from galactose exhibited the shortest lag, whereas those from glucose exhibited the longest lag phase, among the three conditions (Fig. 2c).

### CELLS GROWING IN MELIBIOSE EXHIBIT METABOLIC HETEROGENEITY

Due to the design of the regulatory network dictating melibiose use, the metabolic strategy at a single‐cell resolution is not clear. Hydrolysis of melibiose leads to glucose and galactose being released in the extracellular environment. The resulting monosaccharides each have antagonistic regulatory effect on MEL1. Although galactose acts as an inducer of the system, presence of glucose represses the expression of GAL/MEL pathway even in presence of galactose via the known catabolite repression pathway (Adams 1972; Kew and Douglas 1976; Johnston and Hopper 1982; Post‐Beittenmiller et al. 1984; Torchia and Hopper 1986). MEL1 has an Upstream Activating Sequence (UAS) for induction by Gal4p in presence of galactose and an Upstream Repressible Sequences (URS) for glucose repression mediated by Mig1p (reviewed in Lohr et al. 1995; Rubio‐Texeira 2005). In addition, the actual dynamics of induction and repression of GAL/MEL use pathway is dictated by the ratio of glucose and galactose in the environment (Escalante‐Chong et al. 2015). Moreover, we know that glucose is the preferred carbon source over galactose in the hierarchical sugar use system in yeast (Carlson 1987; Gancedo 1998).

Based on these studies, one plausible strategy for using melibiose at a single cell level in yeast could be that, between the two monosaccharides, an individual cell preferentially uses glucose first. As a result, the genes responsible for galactose use (including MEL1) are repressed. Once the glucose from the media is exhausted, the cell transitions to using galactose. However, use of galactose triggers expression of MEL1p resulting in further hydrolysis of melibiose and release of glucose in the environment. Thus, the cell again switches back to the preferred carbon source, glucose. However, such a strategy involves periodic shifting from one carbon source to another. Due to this asynchrony in the population, there is metabolic heterogeneity at a single‐cell resolution, at any given instant. Moreover, regular transitioning from one carbon source to another requires continuous adjustment in gene expression patterns. This is known to contribute toward fitness costs, in terms of causing a reduction in microbial growth rates (Gorke and Stulke 2008; Lambert and Kussell 2014; New et al. 2014).

As against this, the other possibility of growth in melibiose suggests that during log phase of growth, the population splits into two distinct metabolic states. One fraction of the population uses galactose, and thus releases Mel1p in the environment for hydrolysis. In the remaining fraction of the population, the galactose network is in the OFF state, and cells grow by using the glucose released as a result of melibiose hydrolysis. In such a scenario, at any instant in the population, an individual cell is present in one of the two metabolic states. Such a strategy, where an isogenic population splits into two phenotypic groups, has been observed in both bacteria (Solopova et al. 2014) and yeast (Bagamery et al. 2020), when placed in environments with more than one carbon source.

The heterogeneity in the metabolic state of a population growing in melibiose can be tested by adding 2DG, a nonmetabolizable analog of galactose, to a cellular population. Upon addition, all cells that express Gal1p convert 2DG to a toxic intermediate metabolite (2‐deoxygalactose‐1‐phosphate) and are thereafter killed (Nogi et al. 1984; Platt 1984; Gorman et al. 1992). Only cells that have the GAL system in the OFF state (i.e., have no Gal1p) survive in media containing 2DG. 2DG is transported into the cells via the galactose transporter, Gal2p, and cells grown in presence of 2DG and glucose are not killed (Platt 1984).

**Figure 3:**
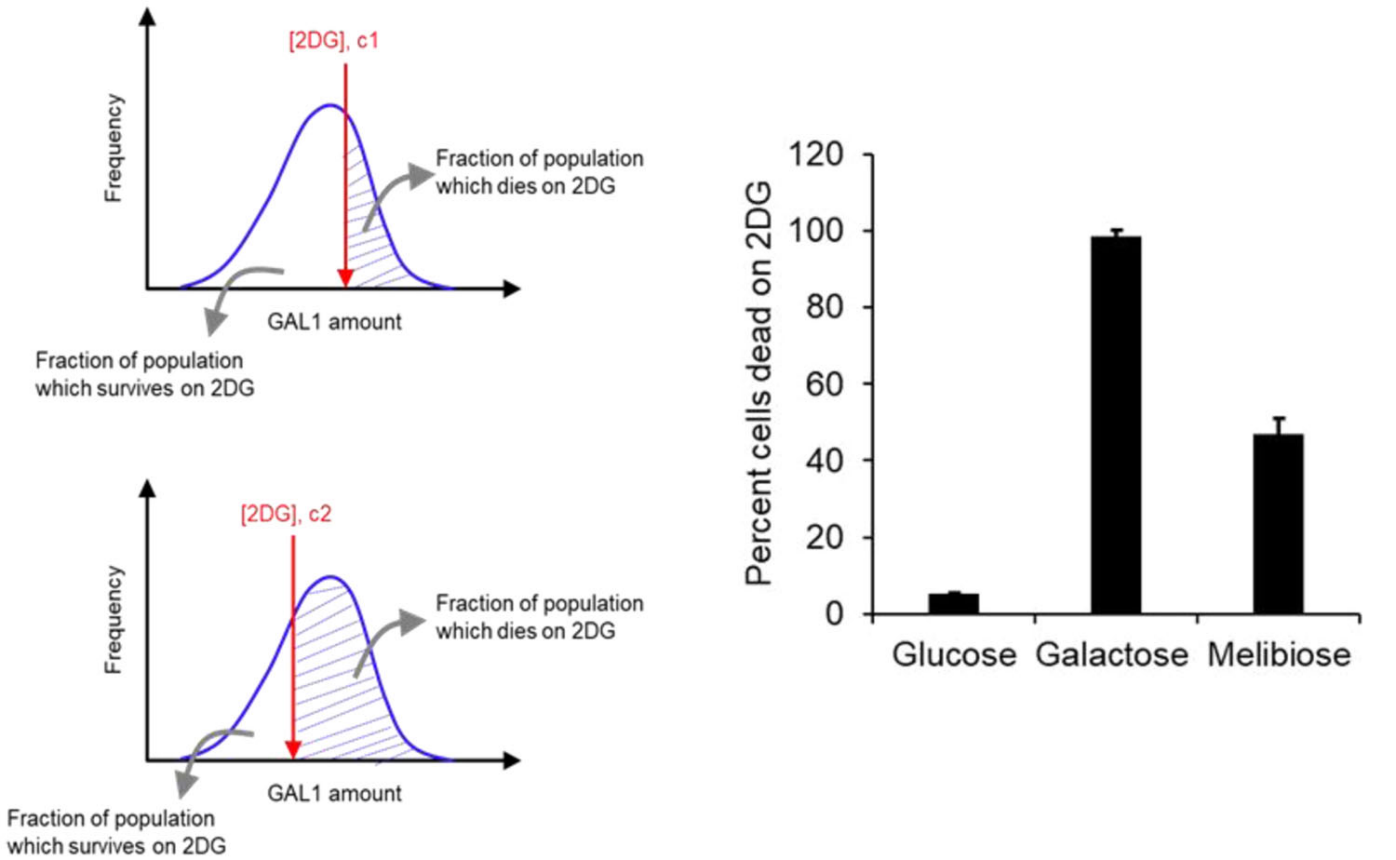
(Left) Imagine a population of *S. cerevisiae* with a distribution of intracellular GAL1 levels. When spread on YPD plates with c1 concentration of 2DG, a GAL1 threshold is placed on the population. No cell with GAL1 level above the concentration survives on this plate. When the identical population is spread on a plate with a higher concentration of 2DG (c2), the GAL1 threshold is lowered. (Right) *Saccharomyces cerevisiae* growing on 2% melibiose exhibits metabolic heterogeneity. This heterogeneity is not exhibited when the population is growing on 2% glucose or 2% galactose. Wild‐type cells were grown to saturation in gly/lac media, and subcultured in respective media to an OD600 of 0.005. Cells were allowed to grow to an of 0.2 and plated on plates containing 2DG. Controls were spread on YPD plates. The experiment was performed in triplicate and the average and standard deviation are reported. The experiment was performed in triplicate. Average and standard deviation are reported.

At the level of a single cell, the population exhibits a distribution of levels of Gal1p expression. Addition of a particular concentration of 2DG (0.3 μM) to the growth media places a threshold in terms of the cellular amounts of Gal1p. Any cell with Gal1p amount greater than this threshold is killed in presence of 2DG. If the concentration of 2DG in the media is increased, the threshold GAL1p amount decreases (Fig. 3). Thus, by studying the survival rates of a population at different 2DG concentrations, we can estimate the Gal1p distribution in that population.

Adding 2DG to cells growing in glucose does not compromise cellular survival (>98% of the cells survive). However, addition of 2DG to cells in the mid‐log phase of growth in galactose leads to nearly 100% cells being killed (Fig. 3). When cells from the mid‐log phase of growth in melibiose are taken and plated on a media containing 2DG, only around 50% of the cells survive. This is compared to almost 100% survival in the lag phase, and almost zero percent survival in the stationary phase of growth, in melibiose. These results demonstrate that during the log phase of growth in melibiose, a population demonstrates metabolic heterogeneity.

**Figure 4:**
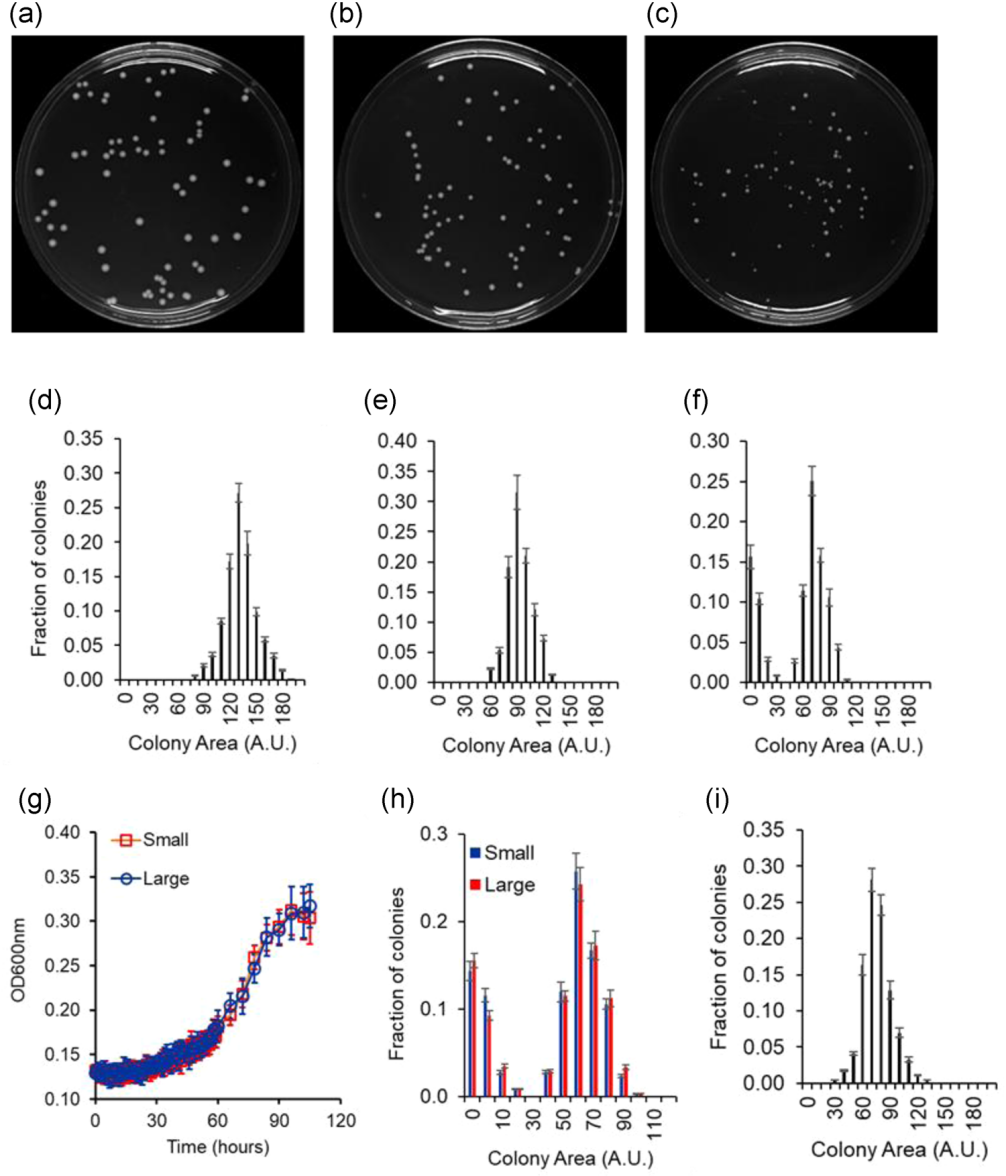
(a–f) Colony size heterogeneity on melibiose plates. Cells grown in gly/lac were plated on plates containing glucose (a), galactose (b), and melibiose (c) as the carbon source. Each sugar was present at a concentration of 2%, and colony size distribution after 60 h of growth is shown. The colony size data were quantified and the distributions are shown for glucose (d), galactose (e), and melibiose (f). Distributions on glucose and galactose plates are represented by a normal distribution. Distribution on melibiose is bimodal. (g) Small and large colonies on melibiose plates exhibit identical growth kinetics, once propagated through gly/lac. Cells from large and small colonies were suspended in gly/lac media and grown to saturation. Cells from the saturated culture were then subcultured in 2% melibiose media to an initial OD600 of 0.005. Growth kinetics of the cultures was then monitored at 30°C. Growth kinetics of six small and six large colonies was analyzed. The average and standard deviation is presented in the figure above. (h) Small and large colonies when passed through gly/lac and thereafter plated on melibiose plates exhibit statistically identical colony size heterogeneity. (i) Colony size heterogeneity is not observed when cells are plated on melibiose from a galactose environment. Wild‐type cells were grown in 2% galactose to saturation and thereafter transferred to a melibiose plates for single colony. After 60 h of growth, size of >500 colonies was measured and the frequency distribution of the size was plotted. All experiments were performed in triplicate, and average and standard deviation are reported.

In addition to the metabolic heterogeneity observed in the liquid media, when plated on melibiose, the population exhibits heterogeneity in the colony size distribution on melibiose. As shown in the Figure 4a–f, the colony size distribution on glucose or galactose plates can be described using a normal distribution. However, the colony size distribution, on plates containing melibiose, is bimodal.

Cells from a large and a small colony were transferred to and grown in gly/lac to saturation. The cells were then transferred to melibiose to a starting OD600 of 0.005 and the kinetics of growth monitored. In such a scenario, the two populations exhibit identical growth kinetics (Fig. 4g). Moreover, when cells from small and large colonies are suspended and grown in gly/lac media to saturation, and thereafter plated on melibiose, they exhibit identical colony size distribution (Fig. 4h). These results demonstrate that the heterogeneity in the colony size on melibiose is phenotypic in nature. As a result, when cells growing in galactose are plated on melibiose, the heterogeneity in the colony size distribution is not observed (Fig. 4i).

### COST‐BENEFIT MODEL PREDICTS THAT POPULATION DIVERSIFICATION CAN BE AN ADAPTIVE STRATEGY IN HIGH‐MELIBIOSE ENVIRONMENTS

Given the metabolic heterogeneity in the ancestral isogenic population in melibiose, we ask the following question: If evolved in melibiose for a few hundred generations, does the metabolic heterogeneity observed collapse or get exaggerated? The two possible outcomes can both be argued as follows.

First, evolution in melibiose can be expected to lead to collapse in the heterogeneity by acquisition of mutation(s), which permit the cell to co‐use glucose and galactose together. If such a generalist has a greater fitness than specialist populations, the population will not diversify genetically. Collapse of hierarchy of sugar use is known to take place even when microorganisms are evolved in precise sugar environments for a prolonged duration (Sievert et al. 2017).

On the other hand, on evolution in melibiose environment, the phenotypic heterogeneity could evolve into a genetic heterogeneity. In such a scenario, the glucose users in the population would evolve to become better adapted to use glucose, whereas the galactose users will evolve to become galactose specialists. Such a genetic split will permit the two genotypes to coexist in the population.

To test the possibility of the two adaptive solutions, we develop a phenomenological mathematical model based on cost‐benefit analysis, which optimizes the growth rate of the culture under the two adaptive solutions. The logic of the model is as follows. In the generalist adaptive solution, mutation(s) permit co‐use of glucose and galactose. To derive benefit from the two sugars, the cell pays a cost to synthesize the necessary enzymes. However, an individual cell cannot process more than a specific amount of carbon flux per unit time. Such constraints are imposed by cellular physiology (Mori et al. 2019).

On the other hand, in the specialist adaptive solution, we hypothesize that a fraction of the population (*x*) uses galactose for growth and splits melibiose. The remaining fraction (1 – *x*) grows on glucose produced as a result of this hydrolysis. Each cell type pays cost and derives benefit in accordance with the carbon source it is using for growth. Because the two metabolic strategies coexist in the solution, during the log phase of growth, the model is solved for the value(s) of *x*, for which the two fractions have an equal growth rate/fitness.

**Figure 5:**
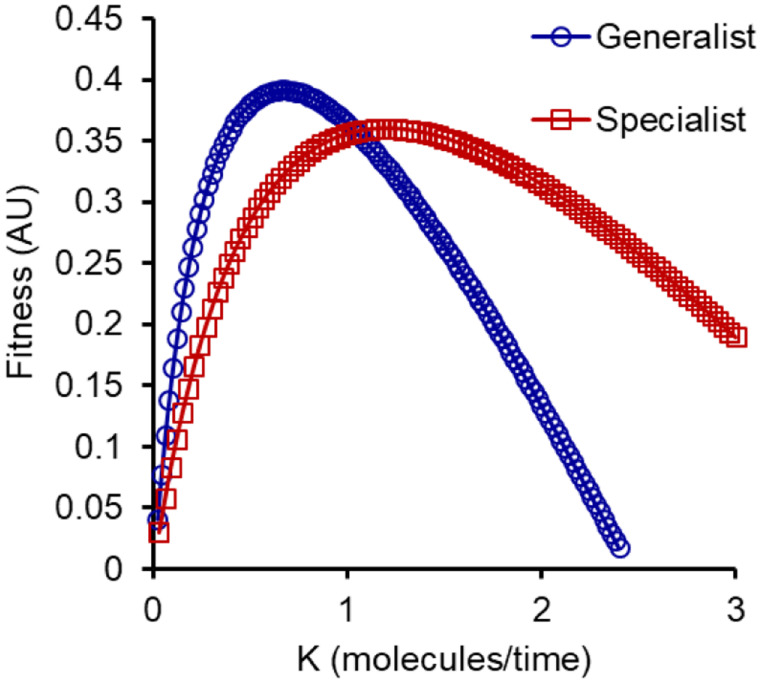
Cost‐benefit model predicts that specialists in glucose and galactose use can evolve in high‐melibiose environments. The *x*‐axis, *K*, equals $\left(\frac{k_{\mathrm{cat}}\;s}{k_{d}}\right)$ and represents the relative rate of melibiose hydrolysis in the surrounding environments.

We then compare the growth rates facilitated by each of the two strategies in the melibiose environment. As shown in the Figure 5, the best growth rate is facilitated by the generalist strategy when the parameter, *p*, $\left(\frac{k_{\mathrm{cat}}\;s}{k_{d}}\right)$ is small. The parameter *p* comprises *k*_cat_, which is the enzyme catalytic activity for MEL1p; *s* is the concentration of substrate melibiose present in the environment; and *k*_d_ is the degradation rate of the MEL1p in the extracellular environment. Thus, overall, the parameter *p* can be seen to be a proxy of the amount of hydrolysis taking place in the extracellular environment per unit time. After a critical value of the resource availability, the specialist strategy confers higher fitness to the population growing in melibiose.

This predicts that at high rates of melibiose hydrolysis, splitting the population into two specialist groups will lead to a higher fitness compared to a generalist strategy. The converse is true when the rate of melibiose hydrolysis is low. We note that the parameters *k*_d_ and *k*_cat_ have not been characterized experimentally, and their values are unknown. In this context, the “high” and “low” concentrations of glucose at which specialists and generalist, respectively, emerge as the optimal strategy are relative in nature.

We assume that the two solutions are accessible in the sequence space (Dutta and Saini 2020). When we pose the specialist groups in the population, we assume that one group exclusively uses glucose and the other uses galactose only. Intermediate strategies are also possible as an adaptive strategy, where one group exhibits a higher propensity for glucose, and the other for galactose. Any such intermediate strategy yields similar results, at different qualitative values.

### EVOLUTION IN HIGH‐MELIBIOSE ENVIRONMENTS

Starting from a diploid ancestor, we evolve yeast populations in melibiose environment for 400 generations in an environment containing 2% melibiose. Three independent lines were evolved as part of the experiment. The evolution experiment was carried so that cells growing in mid‐log phase were transferred from the culture tube to a tube with fresh media. The transfer was thus done at a time in the growth phase when the population exhibited maximum metabolic heterogeneity. We evolved diploids to (a) prevent autodiploidization (Gerstein et al. 2006; Tung et al. 2021) and (b) isolate dominant mutations.

**Figure 6:**
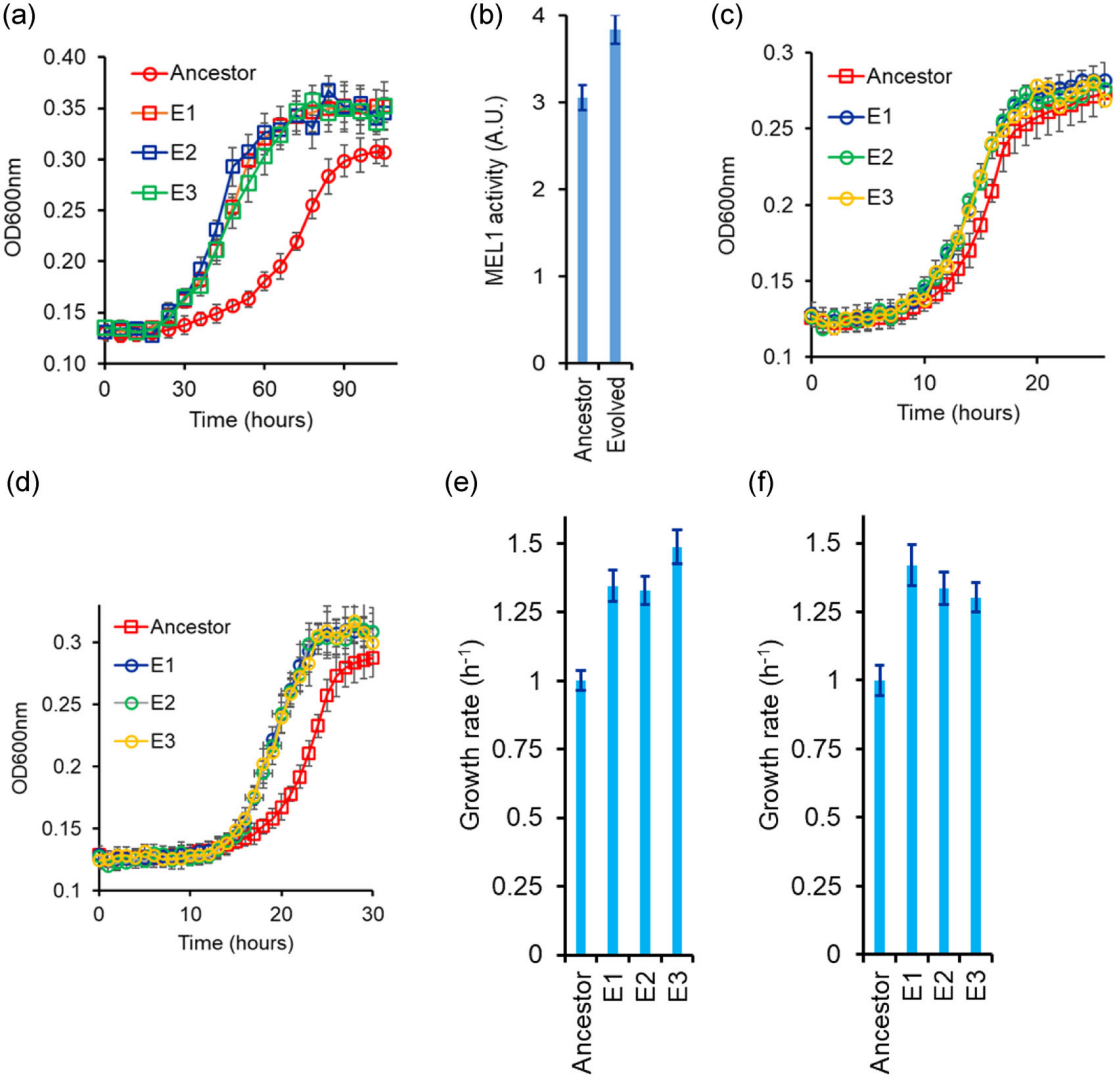
(a) Kinetic of growth in melibiose of the three evolved lines. Cells were grown in gly/lac to saturation and subcultured in 2% melibiose to an initial OD600 of 0.005. Kinetics of growth was monitored at 30°C every 6 h. (b) Evolved strain exhibits a higher MEL1 activity in the supernatant as compared to the ancestor. (c and d) Growth kinetics of evolved lines in glucose (c) and galactose (d). Lines E1, E2, and E3 are the three independently evolved lines. Panels (e) and (f) represent the growth rates in the exponential phase for the three lines for growth in glucose and galactose, respectively. The growth rates of the evolved lines are normalized with respect to the ancestor's growth rate. Line 1 was taken forward for further analysis. All experiments were performed in triplicate, and average and standard deviation are reported.

After evolution for 400 generations, we compare the growth curves for the ancestral and compare that with the evolved lines. The phenotypes tested of all three evolved lines are statistically comparable to each other. As expected, the evolved lines exhibit a short lag phase and a higher growth rate as compared to the ancestor strain (Fig. 6a). Moreover, the evolved lines exhibit a higher MEL1p enzyme activity in the culture media (Fig. 6b).

The beneficial mutations acquired during the evolutionary experiment help the cells exhibit a faster growth in glucose and galactose individually, too. That is, the evolved lines demonstrate a faster growth in glucose and galactose, as compared to the ancestor (Figure 6c–f). This is itself is not a surprising result. Evolution in defined carbon environments with a single carbon source has been demonstrated to have little antagonistic effects (Leiby and Marx 2014). In fact, evolution in one carbon environment has been reported to lead to pleiotropic benefits in other carbon environments too (Choudhury and Saini 2019). On the other hand, antagonism between adaptation on different carbon sources has also been observed, albeit it is less frequently reported (Turner et al. 2015). From the three lines evolved in this experiment, line E1 was further analyzed in this work.

### EVOLUTION FOR 400 GENERATIONS IN 2% MELIBIOSE LEADS TO EXAGGERATED METABOLIC HETEROGENEITY

In a melibiose environment, Gal1p amounts at a single‐cell resolution exhibit a distribution. In this distribution, cells that express high levels of galactose can be termed as “galactose specialists” with respect to their metabolic state. On the other hand, cells with small amounts of galactose can be termed as “glucose specialists” with respect to their metabolic state. The fraction of cells in a population belonging to these two categories can be estimated by adding different concentrations of 2DG to the media, and studying cell survival. When “high” amounts of 2DG are added to media, only cells with minimal/no Gal1p would survive. On the other hand, when “low” amounts of 2DG are added, only cells with high Gal1p amounts would die.

**Figure 7:**
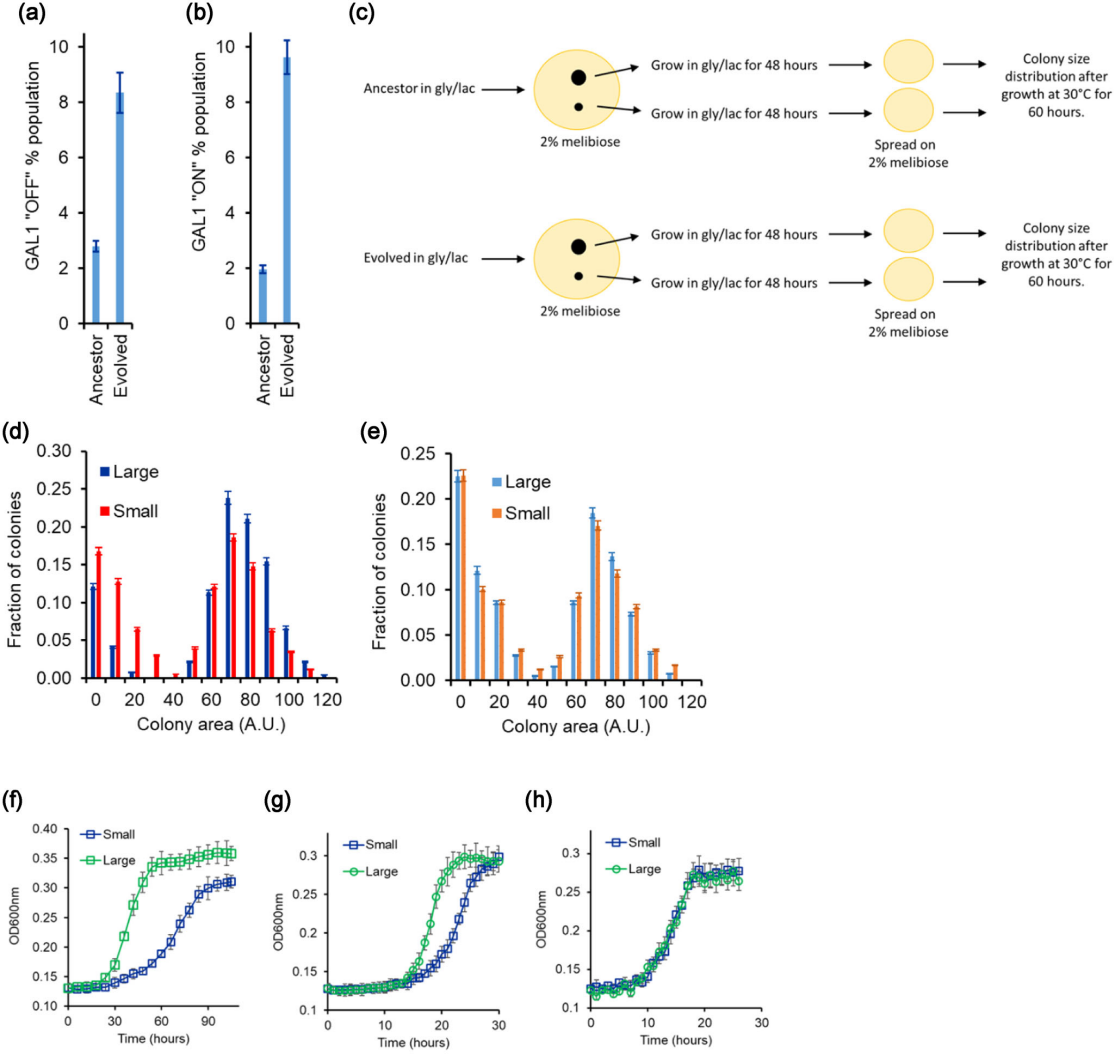
Percent population with low and high GAL1 induction increased in the evolved line (E1). (a) When mid‐log phase culture growing on melibiose was spread on plates with 0.6 μg/mL concentration of 2DG, the evolved line (E1) had roughly fivefold higher percent of population in GAL1 fully induced state. At this concentration of 2DG, only fully induced cells are killed. Percent population was calculated using the fraction of cells that did not survive on plates containing 2DG. (b) When mid‐log phase culture growing on melibiose was spread on plates with 0.3 μg/mL concentration of 2DG, the evolved line (E1) had roughly threefold higher percent of population in GAL1 OFF state. This percent was calculated by counting the CFU on plates containing 2DG versus the CFU on YPD plates. (c) Experimental plan for quantifying colony size distributions for the large and small colonies for the ancestor and the evolved culture. (d) Large and small colonies in the evolved line E1 exhibit statistically significantly different colony size distribution on 2% melibiose plates. (e) Large and small colonies for the ancestor exhibit statistically identical colony size distribution on 2% melibiose plates. More than 1000 colonies were characterized for size for large and small colonies, when plated on melibiose. (f–h) Evolved line E1 when plated on melibiose exhibits small and large colonies. The colonies exhibit different growth kinetics in melibiose (f) and galactose (g). The colonies exhibit similar growth kinetics in glucose (h). All experiments were performed in triplicate, and average and standard deviation are reported.

We add a high concentration of 2DG (0.6 μM) to a culture of the ancestral cells in the mid‐log phase of growth (OD 2) in 2% melibiose. At this concentration, only ∼3% cells survive. We call these cells “glucose specialists,” because they express minimal levels of Gal1p. At the same concentration of 2DG, however, approximately 8% cells of the evolved population were able to survive. Thus, after evolution for 400 generations, the “glucose specialists” in the population increased by almost threefold (Fig. 7a).

To detect galactose specialists, to ancestral population growing in the mid‐log phase in melibiose, we add a low concentration of 2DG (0.3 μM). This concentration only kills cells with fully induced GAL system (when grown in 2% galactose) (Fig. 7b). In the ancestral population, this concentration killed approximately 2% of the population. We call this fraction of the population as “galactose specialists.” In the evolved lines, however, ∼9.5% of the population was killed at this low concentration of 2DG, indicating that the fraction of the population in the metabolic state of “galactose specialists” had increased.

Thus, our results indicate that from the context of phenotypic metabolic state of the cells, both “glucose specialists” and “galactose specialists” percentage in the evolved lines has increased. However, at the concentration of 2DG at which we test this resolution only identifies <10% of the ancestral and <20% of the evolved populations. The remaining population expresses GAL1p amounts in between the two thresholds set by the two 2DG concentrations.

Like the ancestor, the evolved line also exhibits colony size heterogeneity on melibiose plates. To study the differences in the small and large colony phenotype in the evolved line, we pick a small and a large colony on melibiose plate for both ancestor and evolved line E1. Thereafter, we propagate these four colonies through a noninducing nonrepressing media (gly/lac) for ∼15 generations (two 1:100 subcultures). Propagating cells in noninducing nonrepressing conditions for this duration were shown to have removed any differences between the metabolic state of the cells in small and large colonies (Fig. 4).

After this period, the four cultures were plated on melibiose‐containing plates again. The colony size distribution was identical for small and large colonies of the ancestor. However, the colony size distribution from the large and small colonies of the evolved strain was qualitatively different from each other (Fig. 7c).

Moreover, from the glycerol‐lactate media, when the two (small and large, for evolved strain) were tested for their growth on melibiose, the large colony from the evolved culture exhibits a faster growth dynamics in melibiose and galactose, as compared to the small colony (Figure 7d–f). The two exhibit similar growth kinetics in glucose. These results strongly suggest that during the course of the evolution experiment, the original isogenic population split into two groups, and one part of the population evolved to become a galactose specialist.

### SEQUENCING REVEALS MUTATION IN THE GAL3 LOCUS IN THE LARGE COLONIES ON MELIBIOSE COLONIES

**Figure 8:**
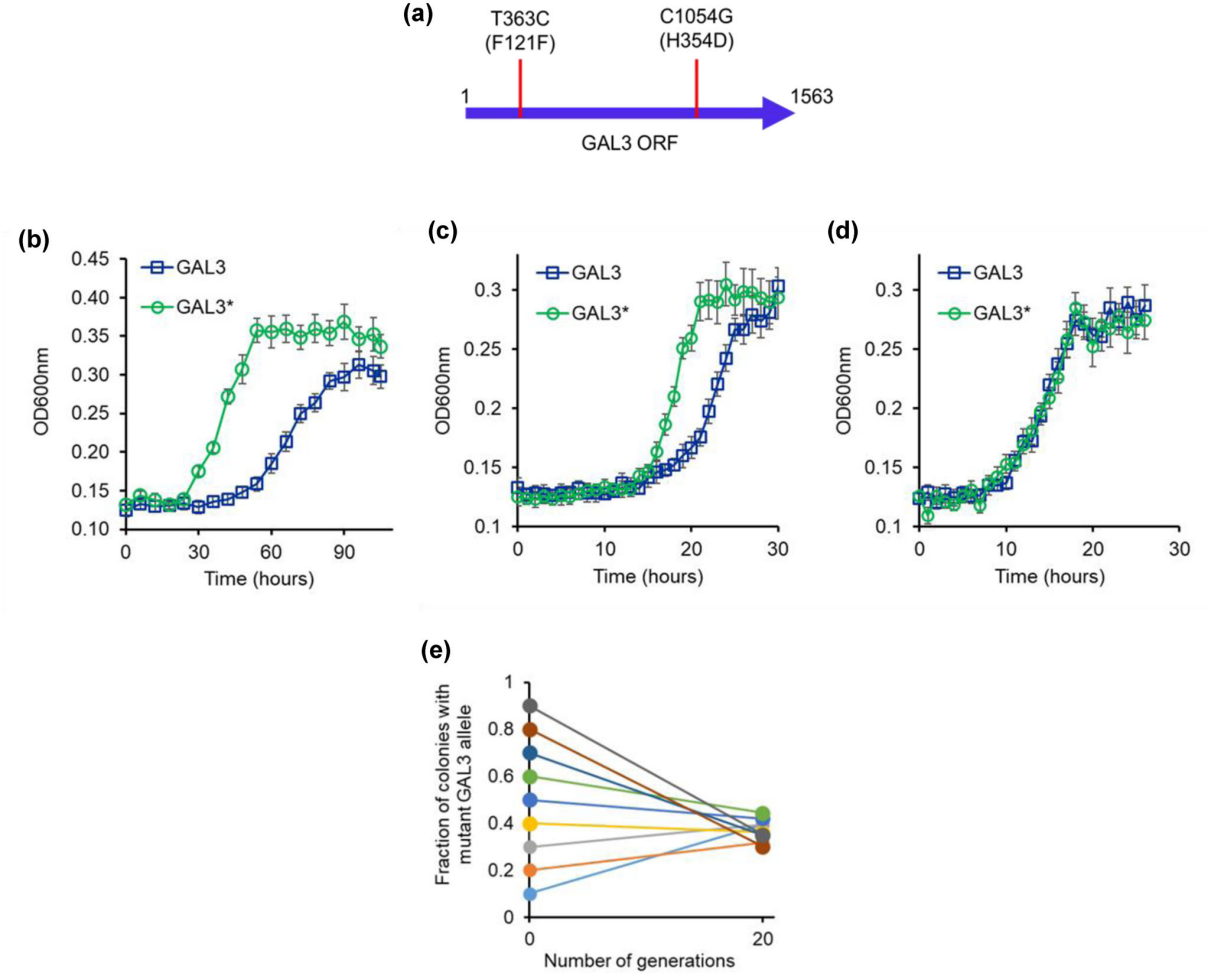
(a) Two SNPs in the GAL3 coding sequence of the large colonies in the line E1. The mutant allele is referred to as GAL3*. L121F was because of a T363C mutation, and H354D was because of a C1054G mutation. The mutation C1054G is found in several yeast isolates and the mutation T363C is present in an environmental isolate NC‐02 (Wang et al. 2015). (b–d) Growth kinetics when the mutant (GAL3*) and the ancestral GAL3 alleles are shifted to the ΔGAL3 strain, and growth kinetics compared in melibiose (b), galactose (c), and glucose (d). All experiments were performed in triplicate, and average and standard deviation are reported. (e) GAL3 and GAL3* haploids grow in melibiose to nearly identical frequencies, independent of the starting frequencies of the two strains. Average of three independent repeats is presented. Standard deviation is less than 0.05.

To determine the genetic basis of the different phenotype of the small and the large colonies in the evolved lines, we sequenced the promoter and the coding regions of the GAL regulon (GAL1, GAL2, GAL3, GAL4, GAL80, and MEL1). For this purpose, the diploids of the small and large colonies were sporulated and dissected to isolate the four haploids from each. All eight haploids so collected were sequenced. Sequencing results revealed that two of the haploids from the large colony had a mutant GAL3 allele (Fig. 8). Previous work has demonstrated that difference in glucose‐galactose signaling was attributed to the alleles present at the GAL3 locus (Lee et al. 2017). As a result, the authors propose that GAL3 is critical for adaptation in glucose‐galactose‐containing media.

The two mutations (T363C and C1054G) lead to one synonymous and one nonsynonymous changes in the coding sequence of GAL3. Interestingly, a recent analysis of GAL3 sequence in environmental isolates of yeast *S. cerevisiae* revealed that these two mutations are also present in the strain NC‐02 (Lee et al. 2017). This particular strain carried two additional mutations in the coding region of GAL3. Moreover, the glucose repression on GAL gene expression in this particular strain was reported to be significantly lower as compared to that in the ancestral sequence (same as the ancestral sequence used in our study).

Gal3p is more than 70% identical with Gal1p; however, it lacks the galactokinase activity of Gal1p (Bhat et al. 1990). Gal3p has a phosphate‐binding loop (spanning residues 156−162). This loop serves as the binding site for the ATP phosphoryl tail. The lack of galactokinase activity of Gal3p has been attributed to the absence of two amino acids in the GLSSSA(A/S)(F/L/I) motif typical of all functional galactokinases. Instead, Gal3p contains the sequence GLSSAF in that position. Gal3p can, however, be converted into a galactokinase through the addition of two amino acids, serine and alanine, after the Serine 164 residue in its coding region (Platt et al. 2000). The two SNPs in the GAL3 coding region encode for a synonymous and nonsynonymous change. Neither of the two SNPs impacts the region of the protein that is close to the active site. However, GAL3 mRNA is known to be degraded faster when glucose is present in the media (Hsu et al. 2012), thus the likely mechanistic change that the mutations cause is not known.

To study the role of the mutant allele, we complemented a ΔGAL3 strain with the ancestral and the mutant GAL3 allele (GAL3*). The growth dynamics measurements from this experiment demonstrate that GAL3* leads to faster induction of the GAL genes, compared to the strain carrying the ancestral GAL3 (Figure 8b–d). Thus, the GAL3* allele isolated from the evolution experiment enhances cellular fitness. In a recent computational study, it was demonstrated that GAL3 mutations lead to tuning of the GAL/MEL system's gene expression dynamics (Rajeshkannan et al. 2022). GAL3 polymorphism has been widely observed in ecological isolates too (Lee et al. 2017). In this context, our results suggest that GAL3 is a mutational target, with respect to evolvability of the GAL/MEL network.

When the two strains (ΔGAL3 carrying GAL3 and ΔGAL3 carrying GAL3*) were co‐cultured at different initial frequencies in a melibiose environment, at saturation, the cultures grew to nearly identical frequencies of the two genotypes (Fig. 8e), thus indicating stable coexistence of the two alleles in the population. In this experiment, as our results demonstrate, the fitness of the participating genotypes is frequency dependent. We speculate that the relative frequency of galactose and glucose users, in a melibiose environment, is highly dynamic, and strongly dependent on the environment and participating genotypes.

### WHY NOT EVOLVE TO BECOME BETTER AT USING MELIBIOSE?

Growth on melibiose is characterized by a relatively long lag phase, when compared to growth rate on monosaccharides glucose or galactose. The delay in growth, when *S. cerevisiae* is growing on melibiose, is due to the slow release of the enzyme α‐galactosidase. The release of the enzyme in the media is faster and higher in the evolved strain, as compared to the ancestor. It is known that compared to the protein coding sequences, promoter regions evolve rapidly (Yona et al. 2018), and in this context, it is surprising that the MEL1 promoter is weakly induced and has little constitutive activity. This is specially so because the fitness gain, in terms of reduced lag time, by increasing the MEL1 promoter strength is significant. Why then is MEL1 promoter so weakly induced? In our work, we do not observe any mutation in the strain where the MEL1 promoter region has acquired any mutation.

**Figure 9:**
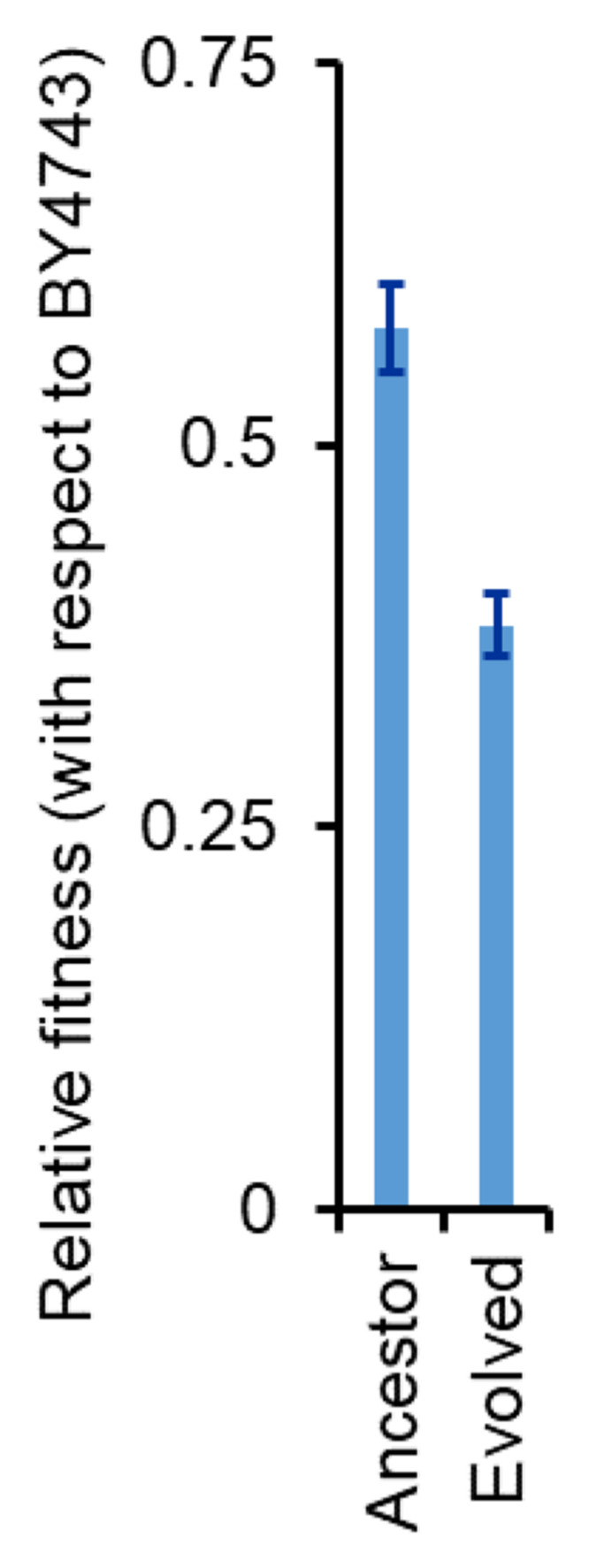
Fitness of the evolved line E1 is lower as compared to that of the ancestor. Competition experiments were performed between BY4743 (a strain lacking *MEL1*) and the ancestor, and between BY4743 and the evolved line E1 in 2% melibiose. Relative fitness was calculated from the comparison of the relative frequency of the two competing strains at the start and end of the competition experiment. The experiments were performed in triplicate, and average and standard deviation are reported.

We hypothesized that a stronger and a faster induction of the α‐galactosidase from the MEL1 promoter makes the strain more susceptible to cheater cells. To test this possibility, we performed competition experiments between the ancestor strain and BY4742 (a strain lacking MEL1). The ΔMEL1 strain (BY4742) is more than 40% fitter as compared to the ancestor strain. However, the fitness of BY4742 was more than 60% higher, when competed with the evolved strain (Fig. 9). This is likely because faster synthesis and release of α‐galactosidase makes the population of *S. cerevisiae* to be more susceptible to exploitation by cheaters and other competitor strains. This disadvantage is likely extremely important in an ecological niche.

A similar phenomenon, where production of a public good is decreased to avoid exploitation by cheaters, has been observed previously. In *Pseudomonas*, when siderophore producers were evolved in the presence of cheater cells, in environments that contained low amounts of iron, the adaptive response of the siderophore producers (co‐operators) was to lower the production of siderophores (O'Brien et al. 2017). The fitness of these evolved co‐operators was lower than that of the ancestor, when grown by themselves. However, in the presence of cheaters, the evolved strains were considerably fitter than the ancestor. Thus, reducing the public good production is an effective strategy in competing against cheater cells.

## Discussion

Isogenic cells exhibit a large variability in metabolic manifestations in steady state conditions. This difference has been attributed to gene expression noise (Nikolic et al. 2017). Darwin proposed that diversification is achieved as specialists emerge to occupy all available niches, big or small, in a given environment (Darwin 1859). Although several examples of a population splitting into two distinct genotypes and stably coexisting thereafter exist (Helling et al. 1987; Friesen et al. 2004; Frenkel et al. 2015; Sousa et al. 2017), little is known about the fundamentals of how this diversification happens (Good et al. 2018).

Such a difference has been observed before—Long Term Evolution Experiment (LTEE)—and was noted in terms of colony size (Rozen and Lenski 2000). The underlying cause of divergence is availability of two resources, where specialization on one leads to reduced fitness on the other. However, in this example, the two resources are made available to the population in a temporal fashion, and are not simultaneously available (Spencer et al. 2007; Plucain et al. 2014). Divergence is also known to occur due to spatial heterogeneity (Spiers et al. 2002; Frenkel et al. 2015).

Antagonism between galactose and glucose is known to occur via glucose‐dependent inactivation of Rgt1p repressor (Johnston and Kim 2005; Santangelo 2006), and galactose‐dependent activation of the Rg1p function, via activation of a co‐repressor Mth1 (Ren et al. 2000). Addition of glucose is also known to degrade GAL3 mRNA, leading to enhanced expression of cyclin Cln3p—a key protein of cell division initiator (Baumgartner et al. 2011). These trade‐offs have been shown to be an essential feature toward driving specialization in sympatric asexual populations (Ostman et al. 2014).

Another dynamic feature of glucose‐galactose use is that, in addition to diauxy, the greatest variability in GAL gene induction is seen when glucose is being consumed slowly, as compared to rapidly (Nguyen‐Huu et al. 2015). In the context of melibiose, because glucose is being released continuously, the rates of glucose depletion in the media are likely quite small. In the context of glucose to galactose transition, cell memory has been shown to be important in dictating the dynamics of transition (Stockwell and Rifkin 2017). Although transition to galactose from noninducing nonrepressing environments like glycerol/lactate is relatively rapid and uniform, transition to galactose from glucose is slow and variable between different cells (Johnston et al. 1994; Lohr et al. 1995; Stockwell et al. 2015).

Several molecular targets could be involved in the diversification. From the context of the galactose network, variation in GAL3 allele alone is able to explain 90% variation in GAL induction kinetics among different environmental isolates (Lee et al. 2017), and GAL3 was shown to be a locus, which modulates the diauxic lag, a selectable trait in the appropriate environmental conditions.

## AUTHOR CONTRIBUTIONS

AM performed experiments, designed experiments, analyzed data, and wrote the manuscript. PN and PA performed experiments. RE performed simulations. SS conceived the study, designed experiments, analyzed data, and wrote the manuscript.

## CONFLICT OF INTEREST

The authors declare no conflict of interest.

## DATA ARCHIVING

There are no data to be archived for the present study.

Associate Editor: R. Kassen

Handling Editor: T. Chapman
